# A retrospective review of a tertiary Hospital’s isolation and de-isolation policy for suspected pulmonary tuberculosis

**DOI:** 10.1186/s12879-014-0547-7

**Published:** 2014-10-14

**Authors:** Shirin Kalimuddin, Jeanne MM Tan, Ban Hock Tan, Jenny GH Low

**Affiliations:** Department of Infectious Diseases, Singapore General Hospital, 20 College Road, Singapore, 169856 Singapore; Department of Internal Medicine, Singapore General Hospital, 20 College Road, Singapore, 169856 Singapore

**Keywords:** Tuberculosis, AFB smear, Isolation

## Abstract

**Background:**

Effective protocols for the isolation and de-isolation of patients with suspected pulmonary tuberculosis (PTB) are essential determinants of health-care costs. Early de-isolation needs to be balanced with the need to prevent nosocomial transmission of PTB. The aim of our study was to evaluate the efficiency of our hospital’s current protocol for isolating and de-isolating patients with suspected PTB, in particular assessing the timeliness to de-isolation of patients with AFB smear negative respiratory samples.

**Methods:**

We retrospectively reviewed 121 patients with suspected PTB who were admitted to our hospital’s isolation ward. We analyzed the time spent in isolation, the total number of respiratory samples that were collected for each patient and the time taken from collection of the first respiratory sample to release of the result of third respiratory sample for acid-fast bacilli (AFB) smear. We also calculated the direct cost of isolation for each patient.

**Results:**

The mean and median number of AFB smears for each patient was three. Thirty percent of patients had four or more AFB smears taken and 20% were de-isolated before the results of three negative AFB smears were obtained. The mean duration of isolation was significantly shorter in patients who had fewer than three negative AFB smears compared to those who had three or more negative AFB smears (three days vs. five days, *p <0.01*). The mean cost in patients who were de-isolated before three negative smears were obtained was USD 947 compared to USD 1,636 in those were only de-isolated after three negative AFB smears (*p <0.01*).

**Conclusions:**

Our study suggests that our institution’s current infection control policy for the isolation of patients with suspected PTB is fairly satisfactory, but may need to be tightened further to prevent true cases of PTB being de-isolated prematurely. However, there may be instances when patients could potentially be de-isolated more quickly without risk to others, thus saving on the use of limited resources and costs to patients.

**Electronic supplementary material:**

The online version of this article (doi:10.1186/s12879-014-0547-7) contains supplementary material, which is available to authorized users.

## Background

Tuberculosis (TB) is a major cause of morbidity and mortality in many countries and a significant health problem worldwide. According to the World Health Organization (WHO), there were more than eight million new cases of TB and approximately 1.4 million deaths due to TB in the year 2011 alone [1]. The incidence rate of TB in Singapore was 6 cases per 100,000 resident population, with over 1600 new cases being reported in 2011 [2]. Although this incidence is the lowest in South-East Asia, it is still several times higher than that in the United States, Western Europe and Australia. Moreover, the incidence of TB in Singapore has been rising since 2008 [3].

Protocols for the isolation of patients with suspected PTB are among the most effective control measures for the prevention of nosocomial transmission of this disease [4]. The AFB smear often provides the first bacteriologic evidence of mycobacterium in a clinical specimen. In addition, smear-positive PTB is deemed more contagious than smear negative PTB [5]. As such, AFB smears of respiratory samples are used by most healthcare facilities to determine when patients with suspected PTB can be removed from isolation. Isolation however, has been shown to impede patient care and negatively impact patient satisfaction [6]. There are also additional costs of personal protective equipment, operation of negative pressure rooms and nursing time [7].

Our hospital has 43 dedicated negative-pressure single-bedded rooms for the isolation of patients with airborne diseases, including those with suspected PTB. In our institution, there is no specific guideline to define such patients, and the decision to isolate is left to the discretion of the managing physician. This often, but not always includes patients (both immunocompetent and immunocompromised, or suspected to be immunocompromised) with chronic respiratory and/or constitutional symptoms such as prolonged cough and loss of weight, and those with chest radiograph findings suspicious of active PTB. Although our institution does not have specific guidelines to determine which patients are at risk for suspected PTB and require isolation, once a decision has been made by the managing physician to isolate a patient, our infection control policy requires them to remain in isolation till three consecutive respiratory samples have been shown to be smear-negative for AFB. For this purpose, spontaneously expectorated sputum, laryngeal swabs and naso-gastric aspirates are obtained from these patients. This is extrapolated from recommendations by the Centers for Disease Control and Prevention and the American Thoracic Society to test three expectorated sputa for mycobacterial culture to exclude infectious TB [8],[9]. These samples however, can take several days to obtain, especially if the patient is unable to co-operate or expectorate spontaneously. The quality of the samples obtained may be suboptimal, leading to reduced diagnostic sensitivity. In some centers, sputum induction has been adopted successfully for increasing the diagnostic yield of PTB. Sputum induction however, may be associated with increased operating costs as it requires special equipment, a dedicated isolation room and trained nurses or physiotherapists to obtain good quality specimens.

The main objective of this retrospective study was to evaluate the timeliness to de-isolation of patients with AFB smear negative respiratory samples. In particular we wanted to assess the excess time AFB smear-negative patients spent in our isolation facility. We hypothesized that AFB smear-negative patients were often kept in isolation rooms longer than necessary. This would subsequently lead to increased hospitalization cost and further stretch the capacity of limited isolation resources.

## Methods

### Settings

The study was carried out in a single institution in Singapore, which is a large 1500 bedded tertiary hospital with over 70,000 patients admitted each year. It has 43 dedicated negative-pressure single-bedded rooms for the isolation of patients with airborne diseases, including those with suspected PTB.

### Study design

This study was conducted as a retrospective review.

### Study population

Patients were included if they met the following criteria:

1. Isolated for suspected PTB between 1st January and 31st December 2010.
2. At least one negative respiratory AFB smear result in the same period.

Patients were excluded if they met the following criteria:

1. Any positive respiratory AFB smear result between 1st January and 31st December 2010.

All patients had respiratory samples (spontaneously expectorated sputum, laryngeal swabs, early morning naso-gastric aspirates or broncho-alveolar lavage samples [BAL]) obtained for fluorochrome and Ziehl-Neelsen staining as well as *Mycobacterium tuberculosis* (*M tuberculosis*) cultures. In our institution, there is no specific algorithm for testing, but in general, spontaneously expectorated sputum collection would be attempted at the first instance. If the patient was unable to co-operate or expectorate, then laryngeal swabs or naso-gastric aspirates would be collected. Patients who were unable to expectorate or co-operate with laryngeal swab or naso-gastric aspirate collection could undergo bronchoscopy to obtain BAL samples according to their managing physician’s preference. A selected number of patients also had respiratory samples evaluated for *M tuberculosis* using polymerase chain reaction (PCR) by the ProbeTec ET DTB assay (Becton-Dickson) at individual physician discretion. Patients would be de-isolated as per institutional protocol if they had three AFB smear negative respiratory samples. In patients with smear positive samples, anti-tuberculous therapy would be commenced and they would remain in isolation for the first two weeks of treatment or until they were discharged home, whichever occurred sooner.

### Selection of study participants

**Figure 1:**
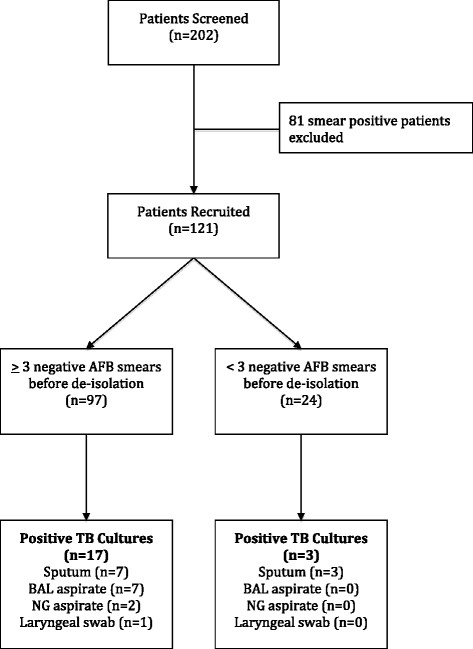
**Flow diagram of patients with AFB smear-negative samples.**

Case records of all patients who were isolated for suspected PTB between 1st January and 31st December 2010 were reviewed. Of these 202 patients, 121 fit our case definition (Figure 1).

### Study variables

Demographic, clinical and radiological data were collected. We analyzed the time spent in isolation, the total number of respiratory samples that were collected for each patient and the time taken from collection of the first respiratory sample to release of the result of third AFB smear. We also analyzed the AFB smear- negative patients whose respiratory samples were subsequently positive for *M tuberculosis* by culture or molecular testing. As the charges for staying in an isolation ward compared to a general ward were greater (USD 290 per day versus USD 260 per day respectively), the cost of each isolation ward stay per patient was also calculated.

### Study investigators

All the case notes were reviewed and analyzed by the authors.

### Ethics approval

Our research was carried out in compliance with the Helsinki Declaration and approval was obtained from our local ethics committee (Singhealth Centralised Institutional Review Board, Reference No: 2013/559/E).

### Analysis plan

A descriptive analysis was performed. Categorical data were described in percentage and numbers. Mean, median and independent sample *T*-test was used for continuous variables, while the Chi-squared or Fisher’s exact test was used for binary variables. A *p-value* of *<0.05* was taken to be significant.

## Results

### Demographics

A total of 121 patients with smear negative respiratory samples were evaluated. Their median age was 62 years. Seventy-eight percent (n = 94) of the subjects were male. Ten percent (n = 12) were foreigners. The majority of subjects lived in high rise public housing (n = 102, 84%), with only three percent (n = 4) living in institutions or dormitories. Nine percent (n = 11) of patients had no fixed abode. Thirty-one percent (n = 38) of the subjects were unemployed while 9.1% (n = 11) were unskilled laborers (Table 1).

**Table 1:** **Demographics of 121 patients with AFB smear negative respiratory samples**

Demographic	
Age - yr	
Median (range)	62 (16–88)
Sex - no. (%)	
Male Residency - no. (%)	94 (77.6))
Singapore Residents	109 (90)
Foreigners/Immigrants	12 (10)
Type of Housing - no. (%)	
Public housing	102 (84.3)
Private Housing	4 (3.3)
Institutions or dormitories	4 (3.3)
No fixed abode	11 (9.1)
Occupation - no. (%)	
Unemployed	38 (31.4)
Managerial/Professional	5 (4.1)
Skilled Labor (Non-managerial/professional)	10 (8.3)
Unskilled labor	11 (9.1)
Unknown	50 (41.3)
Others	7 (5.8)

### Clinical and radiological data

Of the 121 patients, almost a quarter (n = 27) had a prior history of TB. Sixty percent (n = 73) were lifetime non-smokers. Two-thirds of patients (n = 79) had co-morbidities such as hypertension, diabetes mellitus, hyperlipidemia, ischemic heart disease, renal failure, malignancy or chronic lung disease. Twenty percent of these patients had three or more co-morbidities. Hypertension was the most common co-morbidity, followed by diabetes mellitus. Six patients were infected with HIV (Table 2).

**Table 2:** **Clinical and radiological characteristics of 121 patients with AFB smear negative respiratory samples**

Characteristics	
Co-morbidities - no. (%^#^)	
Previous history of TB	27 (22.3)
Current or ex-smoker	48 (40)
Hypertension	28 (23.1)
Diabetes mellitus	23 (19)
Hyperlipidemia	19 (16.1)
Ischemic heart disease	15 (12.4)
Renal failure	13 (10.7)
Chronic lung disease	4 (3.3)
Malignancy	12 (9.9)
HIV	6 (4.9)
Presenting Symptoms - no. (%^#^)	
Cough	40 (30.3)
Fever	20 (16.7)
Anorexia	14 (12)
Loss of weight	20 (16.3)
Dyspnea	17 (13.8)
Hemoptysis	9 (7.6)
Radiological Findings - no.(%^#^)	
Abnormal CXR	116 (95.8)
Consolidation	40 (33)
Nodules	21 (17)
Cavitation	10 (8.2)
Other abnormality*	45 (37.2)

TB = tuberculosis, HIV = human immunodeficiency virus.

^#^percentages do not add up to 100% as each patient may have more than one co-morbidity, presenting symptom or abnormal radiological finding.

*includes granuloma, pleural effusion and mass lesions.

Ninety percent of patients who were isolated were symptomatic - with more than half of these patients suffering from chronic cough or fever. Other symptoms included anorexia (27%), loss of weight (16.3%), dyspnea (13.8%) and hemoptysis (7.6%) (Table 2).

The median duration patients were symptomatic for any of the following: cough, fever, anorexia, loss of weight, dyspnea and hemoptysis, was two weeks (mean: 46 days, range: 1–365 days). Ninety-six percent of patients had an abnormal chest radiograph. The most common abnormality being consolidation in two-thirds (n = 40). Nodules were present in 17% (n = 21) and 37.2% (n = 45) had other abnormalities such as pleural effusion, interstitial infiltrates, granuloma, and mass lesions. Less than ten percent of patients had cavitatory lesions. Twelve percent (n = 15) of radiographs were reported as showing radiological changes suspicious of active PTB (Table 2).

### Management and diagnosis

There were a total of 376 respiratory specimens collected from 121 patients. The most common respiratory sample obtained was sputum (63%), followed by laryngeal swabs (20%), BAL specimens (10%) and naso-gastric aspirates (7%). The mean and median number of smears for each patient was three. Thirty-six patients (30%) had four or more smears performed despite hospital protocol requiring only three samples. Twenty-four patients (20%) were de-isolated prematurely before the results of three negative AFB smears were obtained (Figure 1).

As to be expected, the mean duration of isolation was significantly shorter in patients who had fewer than three negative smears compared to those who had three or more negative smears (three days vs. five days, *p <0.01*). The overall mean cost of isolation to each patient was USD 1,440 (range: USD 290 - USD 5,510). The mean cost in patients who were de-isolated before three negative smears were obtained was USD 947 compared to USD 1,636 in those were only de-isolated after three negative smears (p *<0.01*).

Of the patients who followed hospital protocol requiring at least three negative smears before de-isolation, the mean duration from collection of the first smear to reporting of the third negative smear was 3.8 days (median: 3 days, range: 0–22 days). On average, the time taken from result of the third negative smear to de-isolation was 2.2 days but could range from as short as 0 to as long as 11 days. Thirty-two patients (26.4%) remained in isolation for more than 24 hours after the result of the third negative smear was made available.

A total of 20 out of 121 patients (16.5%) were subsequently diagnosed with PTB based on positive culture results for *M tuberculosis,* despite having AFB smear-negative respiratory samples*.* Half of these were from sputum samples, 35% from BAL fluid while the remaining 15% were from naso-gastric aspirate or laryngeal swabs (Figure 1).

Thirteen patients (10.7%) had PCR for *M tuberculosis* performed on their sputum or BAL samples at their managing physician’s discretion. Of these 13, four had positive PCR results (only two of these four patients subsequently had cultures that returned positive for TB).

Of the 20 patients who had positive TB culture results, 19 (95%) were symptomatic for cough, fever, hemoptysis, dyspnea, anorexia or loss of weight. This was compared to 88% (n = 89) of patients in the group who were culture negative. The difference however was not statistically significant. The median duration of symptoms was 21 days in the culture positive group and 14 days in the culture negative group (*p = 0.634*). Twenty-five percent (n = 5) of culture positive patients had chest radiographs reported as being suspicious for active TB compared with ten percent (n = 10) of culture negative patients although this difference was not statistically significant. The mean duration of hospitalization was longer in patients with a positive culture although this did not reach statistical significance (18.4 versus 11.6 days, *p = 0.057*). The mean cost incurred from utilization of isolation beds was significantly greater in those with a positive culture compared to those who were culture negative (USD 2624 versus USD 1727, ***p < 0.01***) (Table 3). This cost was computed directly from cost per day per room multiplied by total days spent in isolation room per patient.

**Table 3:** **Comparison of TB culture positive and culture negative patients**

	Positive TB Culture (N = 20)	Negative TB Culture (N = 101)	p-value
Mean Age - yr	58.4	60.9	0.525
Symptomatic* - no. (%)	19 (95)	89 (88.1)	0.692
Median symptom duration - days	21	14	0.634
CXR suggestive of active TB - no. (%)	5 (25)	10 (10)	0.129
Mean length of hospitalization - days	18.4	11.6	0.057
Mean cost of stay in isolation ward - USD	2624	1727	<0.01

CXR = chest radiograph, TB = tuberculosis.

*includes cough, fever, anorexia, loss of weight, dyspnea and hemoptysis.

Of the 24 patients who were de-isolated prematurely, three patients were subsequently diagnosed with PTB based on positive TB culture, giving an incidence of 12.5% TB positivity rate compared to 17.5% in the group who were de-isolated only after three negative AFB smears. None of the three patients had chest radiograph findings suspicious of active PTB.

Three patients died during their stay in isolation. All three patients had been diagnosed with PTB but none of the deaths were directly attributed to PTB.

## Discussion

In this study, we evaluated the efficiency of our hospital’s current protocol for isolating and de-isolating patients with suspected PTB. In particular, we assessed both the timeliness of de-isolating patients who were AFB smear-negative, as well as the effectiveness of resource utilization by evaluating the direct hospitalization cost only.

Based on CDC guidelines, our institution’s infection control policy requires patients to have at least three negative sputum AFB smears before they can be de-isolated [8]. Our study found that 20% (n = 24) of patients were de-isolated prematurely before three negative AFB smears were obtained. None of these patients had chest radiograph features suggestive of PTB and only three patients of these 24 (12.5%) were subsequently found to have culture positive PTB. To our knowledge, there have been at least three studies which have examined the sensitivity of consecutive smears. Nelson et al. [10] found that only 13% of the third samples were positive when the first two smears were negative, while Siddiqui et al. [11] reported a similar rate of 11%. A meta-analysis by Burken et al. [12] concluded that the sensitivity of two AFB smears was the same as that of three AFB smears for the diagnosis of PTB. There have also been studies which suggested that pre-test probability of PTB may be determined by evaluating risk factors [13]-[15]. A systematic review by Wisnivesky et al. [16] likewise suggested prediction rules incorporating risk factors such as chronic symptoms, fever and upper lobe abnormalities on chest radiograph to identify those with a low risk of PTB. The findings from our study, together with evidence from others, suggests that it is safe to de-isolate patients once two negative smears are obtained, especially if the patient’s pre-test probability for PTB is low. In our study we found that the financial cost to patients was less in those who were de-isolated before three negative AFB smears, compared to those who were only de-isolated after three negative AFB smears were obtained. It is perhaps not unreasonable to consider earlier de-isolation to strike a right balance between the need for isolation to protect public health interests and early de-isolation for low risk patients to optimize scarce isolation resources. Such a measure may also potentially translate into cost savings for individual patients without compromising public health at large. In our country where healthcare cost is mainly borne by the individual, such cost reductions would substantially reduce out-of-pocket expenses for the patient. We do note however that our calculation of the cost of isolation only included isolation bed charges and did not take into account other costs such as charges for human resources and indirect costs. This is a limitation of the retrospective nature of our study. It is highly conceivable that the true cost is much higher than reported in this study.

A third of patients in our study were only de-isolated after four or more negative AFB smears were obtained. Results from previous studies have shown that sensitivity of diagnosing PTB beyond three negative AFB smears is not increased [11],[12],[16]. Thus, in one-third of our patients, there was unnecessary usage of limited resources and extra cost incurred through additional testing. We did not specifically analyze the reasons for these extra tests. However we postulate that this may have been a result of communication between healthcare workers and/or a lack of co-ordination in the handling of samples. Further efforts to improve intra-hospital work processes so as to reduce wastage should be considered in view of our findings.

CDC guidelines estimate that it should take no longer than two days to safely de-isolate a patient following their protocol. We found however that the average time taken from obtaining the results of three negative AFB smears to de-isolation of our patients took at least five days. Many others have looked at the utility of induced sputum to reduce time needed for collection of three AFB smears samples [17],[18]. Inducing sputum allows all three samples to be collected within 24 hours, independent of patient’s ability to expectorate. A patient’s isolation ward stay could potentially be reduced to one day. Compared to the extra cost of isolation stay of between USD 30 and USD 570 for a patient using our current protocol for respiratory sample collection, induced sputum would potentially translate into direct cost savings of up to USD 540. This cost saving remains even after factoring the extra charge of USD 40 for the procedure. Our institution is currently in the process of setting up such a service and we anticipate that such a service would greatly reduce time from sputum collection to safe de-isolation.

## Conclusions

Our study suggests that our institution’s current infection control policy for the isolation of patients with suspected PTB is fairly satisfactory, but may need to be tightened further to prevent true cases of PTB being de-isolated prematurely. However, there may be instances when patients could potentially be de-isolated more quickly without risk to others, thus saving on the use of limited resources. We predict that the implementation of our sputum induction service will aid in more rapid de-isolation of patients.

## Authors’ contributions

SK participated in the design of the study, collected the data and drafted the manuscript. JMMT performed the statistical analysis. BHT provided overall guidance and helped review the manuscript. JGHL helped in conception of the study, participated in the study design and helped draft the manuscript. All authors read and approved the final manuscript.

## Authors’ original submitted files for images

Below are the links to the authors’ original submitted files for images. Authors’ original file for figure 1 Authors’ original file for figure 2 Authors’ original file for figure 3
