# Supplementary figures and images for: A retrospective review of a tertiary Hospital’s isolation and de-isolation policy for suspected pulmonary tuberculosis
Source: BMC Infect Dis. 2014 Oct 14;14:547. doi: 10.1186/s12879-014-0547-7 (PMC4197325; doi:10.1186/s12879-014-0547-7)

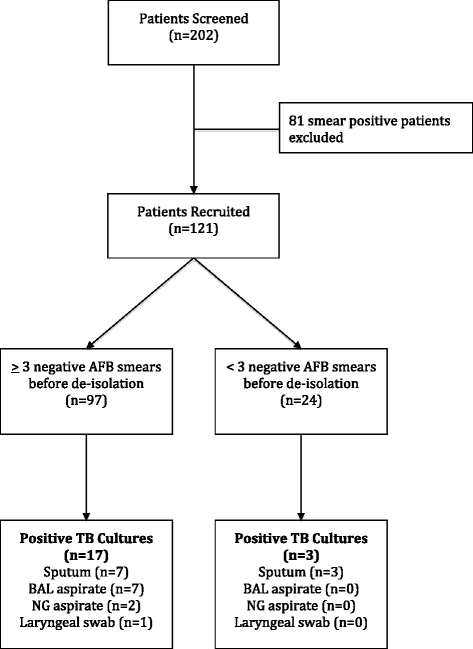

Supplement: Supplementary file 1 — Authors’ original file for figure 1 [file 12879_2014_547_MOESM1_ESM.gif]
