# Supplementary material for: A retrospective review of a tertiary Hospital’s isolation and de-isolation policy for suspected pulmonary tuberculosis
Source: BMC Infect Dis. 2014 Oct 14;14:547. doi: 10.1186/s12879-014-0547-7 (PMC4197325; doi:10.1186/s12879-014-0547-7)
Supplement: Supplementary file 2 — Authors’ original file for figure 2 [file 12879_2014_547_MOESM2_ESM.docx]

| **Characteristics** |  |
| --- | --- |
| Co-morbidities – no. (%) |  |
| Previous history of TB | 27 (22.3) |
| Current or ex-smoker | 48 (40) |
| Hypertension | 28 (23.1) |
| Diabetes mellitus | 23 (19) |
| Hyperlipidemia | 19 (16.1) |
| Ischemic heart disease | 15 (12.4) |
| Renal failure | 13 (10.7) |
| Chronic lung disease | 4 (3.3) |
| Malignancy | 12 (9.9) |
| HIV | 6 (4.9) |
| Presenting Symptoms – no. (%) |  |
| Cough | 40 (30.3) |
| Fever | 20 (16.7) |
| Anorexia | 14 (12) |
| Loss of weight | 20 (16.3) |
| Dyspnea | 17 (13.8) |
| Hemoptysis | 9 (7.6) |
| Radiological Findings – no.(%) |  |
| Abnormal CXR | 116(95.8) |
| Consolidation | 40(33) |
| Nodules | 21(17) |
| Cavitation | 10(8.2) |
| Other abnormality* | 45(37.2) |
