# Supplementary material for: A retrospective review of a tertiary Hospital’s isolation and de-isolation policy for suspected pulmonary tuberculosis
Source: BMC Infect Dis. 2014 Oct 14;14:547. doi: 10.1186/s12879-014-0547-7 (PMC4197325; doi:10.1186/s12879-014-0547-7)
Supplement: Supplementary file 3 — Authors’ original file for figure 3 [file 12879_2014_547_MOESM3_ESM.docx]

|  | Positive TB Culture (N=20) | Negative TB Culture (N=101) | p-value |
| --- | --- | --- | --- |
| Mean Age – yr | 58.4 | 60.9 | 0.525 |
| Symptomatic* - no. (%) | 19 (95) | 89 (88.1) | 0.692 |
| Median symptom duration - days | 21 | 14 | 0.634 |
| CXR suggestive of active TB – no. (%) | 5 (25) | 10 (10) | 0.129 |
| Mean length of hospitalization - days | 18.4 | 11.6 | 0.057 |
| Mean cost of stay in  isolation ward - USD | 2624 | 1727 | **<0.01** |
